# Current Hospital Topics

**Published:** 1906-11-17

**Authors:** 


### HOSPITAL ADMINISTRATION.

### CONSTRUCTION AND ECONOMICS.

### CURRENT HOSPITAL TOPICS.

#### Mr. Pietro J. Michelli, C.M.G.

His Majesty King Edward VII. has made Mr. P. J. Michelli the able Secretary of the Tropical School of Medicine and the Dreadnought Seamen's Hospital a Companion of the Most Distinguished Order of St. Michael and St. George. We believe there is not a single worker in the hospital world of London who will not rejoice to hear that Mr. Michelli has been thus honoured by His Sovereign. Mr. Michelli has worked hard for many years and has always displayed much enthusiasm, ability and tact in the discharge of his multifarious and onerous duties. He has never spared himself, or had a thought apart from his work for the Dreadnought, and the Tropical and London Clinical Schools of Medicine. The success which has attended these Institutions for years past has been largely due to Mr. Michelli's indomitable perseverance. He has well earned the honour just conferred upon him and we hope he may live many years to enjoy it with the wider opportunities for further work which lie before him in the future.

#### Grimsby and District Hospital.

There is a keenness about the interest taken in the management of many provincial hospitals which is stimulating to workers in greater cities. In the provinces the hospital is a centre of general interest and activity, and the higher offices connected with it are sought with diligence by the leading citizens. After seven years' service Mr. C. J. Carter has resigned the chairmanship of this hospital and Mr. J. P. Wintringham, for many years honorary secretary, has been elected in his place. Mr. Carter has served the hospital in various capacities for some twenty years, and during his chairmanship it is claimed that he has converted an old into a modern hospital, with the warm support and cooperation of the committee and his fellow townsmen. Mr. Carter, though much occupied in business, devoted a great deal of time to the work of the hospital, and all its supporters, from the president downwards, regret his resignation and highly appreciate his services. Mr. Carter has worked well with the medical and nursing staffs, whose interests, with those of the patients, were his chief consideration. In recognition of Mr. Carter's good work he has been unanimously recommended for election to the office of vice-president.

#### Central London Throat, Nose, and Ear Hospital.

We are reminded by the invitation to attend the opening of a new wing of this hospital by H.R.H. Princess Louise on the 19th instant, that Mr. Richard Kershaw, the secretary, has devoted thirty years of his life to the interests of this institution. The average number of persons admitted to treatment in the decade ending 1885, compared with the decade ending 1905, has been as follows: The In-patients have increased from an average of 905 to 3,400; the out-patients have increased from an average of 33,293 to 90,717; and the total attendances of patients have increased from an average of 175,000 to upwards of 510,000. The hospital was commenced in 1874 with an out-patient department only. Four years later it made provision for sixteen in-patients in addition. From that time to the present the in-patient accommodation has not been increased owing to a lack of funds, and the same cause has prevented the committee carrying out a larger building scheme, which would have given the hospital thirty-two beds. The new building about to be opened will cost some £7,000, and comprises an operation theatre, an electric lift, and four wards, one of which is for children. No one who has thoroughly examined the work which has been going on at this hospital for upwards of thirty years could fail to have been struck with the excellence of most of the arrangements and the thoroughness and method which has characterised the administration. That is why, no doubt, the Central London Throat and Ear Hospital is so popular with its patients, all of whom have the privilege of paying according to their means, though not a remunerative rate, and care is taken to prevent hospital abuse. We should like to see one great throat and ear hospital for the whole of London, and we look forward to the time when the present negotiations for amalgamating all the throat hospitals will be crowned with success. There are many difficulties to be overcome, but good progress has been made, and we are glad to notice a spirit of conciliation abroad, without which any amalgamation scheme must necessarily fail. Mr Richard Kershaw is entitled to the thanks, not only of the governors, but of the tens of thousands of patients who have passed through his hands during the thirty-two years of this hospital's existence.

#### The Uniform System of Accounts in the United States.

We are gratified to be able to announce the successful completion of the work of the committee appointed at the seventh annual conference of the Association of Hospital Superintendents, Boston, U.S.A., to prepare a uniform system of accounts for hospital purposes. The system has been carefully elaborated, and it is very full and complete as it stands. It has already been adopted by four of the most important hospitals in New York—*i.e.*, the New York Hospital, the Presbyterian Hospital, the Roosevelt Hospital, and St. Luke's Hospital. It is interesting to note that, although the system formulated is very full, and in some respects elaborate, the experience of the Presbyterian Hospital shows that it does not require more than one man's time to keep the whole of the books, and to carry out the system completely. It will interest British hospital authorities to know that the expenditure is classified under the following main heads: administration expenses; professional care of patients; departmental expenses, in which last are included ambulance, pathological, laboratory, training school, housekeeping, kitchen, laundry, and steward's department. Under the latter head all provisions are included. Then we have general house and property expenses, including lighting, insurance, rent, fuel, water, and incidental repairs. The income is divided up into hospital receipts derived from paying patients, special nursing, ambulance fees, etc., and then other revenue, which includes contributions from voluntary sources and grants from the public treasury, as well as legacies and interest on investments. Then there is a capital account giving a summary of the financial transactions for a year, then a comparative balance sheet for the two previous years, and a statement showing the increase or decrease in the capital funds under various heads. The new system also includes comparative statistics for two previous years of all admissions to treatment and discharges given under the head of each set of wards according to the ward units. Then the beds in the hospital are summarised under free beds, endowed beds, pay wards and private rooms, and the percentage which each bears to the total, together with the total patient days' treatment, and the average patients per day, with the daily average cost of each class of patient, are set forth. Finally there is a patient's table giving the same information about the out-patient work done by the hospital under the headings emergency ward, dispensary, ambulance, and visiting or home nursing. The arrangements and form of statistics given concerning the patients treated are a great improvement upon anything we have yet seen, and we commend them to the attention of the managers of British hospitals. A schedule of these particulars will be found on pages 33 and 34 of the *National Hospital Record* for September, or a pamphlet describing the system could no doubt be obtained on writing to Dr. Fisher, medical superintendent, Presbyterian Hospital, New York, to whose persistent energy the formulation of a uniform system of accounts for American hospitals is largely due.

